# Chemical Display of Pyrimidine Bases Flipped Out by Modification-Dependent Restriction Endonucleases of MspJI and PvuRts1I Families

**DOI:** 10.1371/journal.pone.0114580

**Published:** 2014-12-08

**Authors:** Evelina Zagorskaitė, Giedrius Sasnauskas

**Affiliations:** Department of Protein–DNA Interactions, Institute of Biotechnology, Vilnius University, Vilnius, Lithuania; New England Biolabs, Inc., United States of America

## Abstract

The epigenetic DNA modifications 5-methylcytosine (5mC) and 5-hydroxymethylcytosine (5hmC) in eukaryotes are recognized either in the context of double-stranded DNA (e.g., by the methyl-CpG binding domain of MeCP2), or in the flipped-out state (e.g., by the SRA domain of UHRF1). The SRA-like domains and the base-flipping mechanism for 5(h)mC recognition are also shared by the recently discovered prokaryotic modification-dependent endonucleases of the MspJI and PvuRts1I families. Since the mechanism of modified cytosine recognition by many potential eukaryotic and prokaryotic 5(h)mC “readers” is still unknown, a fast solution based method for the detection of extrahelical 5(h)mC would be very useful. In the present study we tested base-flipping by MspJI- and PvuRts1I-like restriction enzymes using several solution-based methods, including fluorescence measurements of the cytosine analog pyrrolocytosine and chemical modification of extrahelical pyrimidines with chloroacetaldehyde and KMnO_4_. We find that only KMnO_4_ proved an efficient probe for the positive display of flipped out pyrimidines, albeit the method required either non-physiological pH (4.3) or a substitution of the target cytosine with thymine. Our results imply that DNA recognition mechanism of 5(h)mC binding proteins should be tested using a combination of all available methods, as the lack of a positive signal in some assays does not exclude the base flipping mechanism.

## Introduction

**Figure 1:**
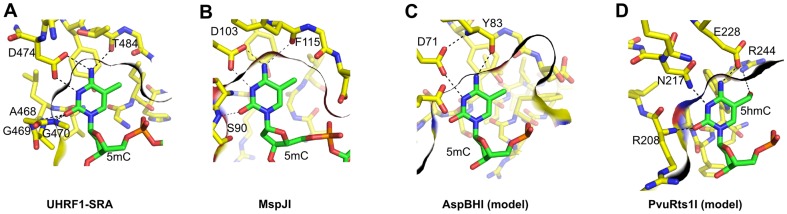
The modified cytosine binding pockets. (A-B) 5-methylcytosine recognition by the UHRF1 SRA domain (PDB ID 3fde) and the DNA recognition domain of MspJI endonuclease (PDB ID 4r28). The indicated protein pocket residues make base-specific contacts to the extrahelical base [19], [45]. (C-D) The models for the modified cytosine recognition by the DNA binding domains of AspBHI (PDB ID 4oc8) and PvuRts1I (PDB ID 4oq2, see Materials and Methods for details). The indicated amino acid residues could form base-specific contacts to the extruded base. In the case of AspBHI, this would require protonation of the D71 residue. In each panel the dark line marks the boundaries of the protein pocket cut at the plane of the cytosine ring.

5-methylcytosine (5mC) and 5-hydroxymethylcytosine (5hmC) are important epigenetic modifications of mammalian and plant DNA. The methylation and hydroxymethylation levels are dynamic and vary in different types of cells during development, differentiation, aging, and disease [1], [2]. Structural studies of eukaryotic 5mC/5hmC binding domains revealed two different strategies for the modified base recognition. Proteins that share the methyl-CpG binding domain, including MBD1, MBD2, MBD4, and MeCP2, also a zinc-finger protein Kaiso, recognize modified cytosine in the context of a Watson-Crick base pair [3]–[7]. In contrast, the SRA (SET and RING-associated) domains of UHRF1, UHRF2, and SUVH5 proteins flip out the modified base and place it in a protein pocket [8]–[12] (Fig. 1A).

In prokaryotes modification-dependent restriction endonucleases protect host bacteria from bacteriophages containing modified DNA. The McrBC complex recognizes 5mC, 5hmC or 4-methylcytosine-containing sequences, and cleaves DNA at a variable position from the recognition site. The crystal structure of the McrBC DNA binding domain (McrB-N) revealed that despite an unrelated tertiary structure, it follows the same mechanism for modified base recognition as the SRA domains: the base is flipped out into a protein pocket [13]. MspJI-like enzymes recognize 5mC and 5hmC modifications in various sequence contexts (for example, 5′-5mCNNR-3′ for MspJI) and cleave top and bottom DNA strands 12/16 nucleotides downstream of the modified base. PvuRts1I family enzymes recognize DNA substrates with 5hmC or glucosylated 5hmC (5ghmC) modifications and, unlike other modification-dependent enzymes, discriminate against substrates with 5mC [14], [15]. Due to strict specificity for unmodified DNA and fixed cleavage positions, both MspJI- and PvuRts1I-like enzymes are used as molecular tools for single-base resolution mapping of 5mC and 5hmC modifications in eukaryotic genomes [16], [17]. The recently solved structures of MspJI, AspBHI (MspJI family), PvuRts1I and AbaSI (PvuRts1I family) enzymes [18]–[23] revealed that these enzymes are comprised of a PD-(D/E)XK nuclease domain fused to a SRA-like DNA binding domain (DBD). The co-crystal structure of the MspJI-DNA complex demonstrated that MspJI flips-out 5-methylcytosine into a protein pocket where a set of base-specific contacts are made (Fig. 1B) [21]. Mutational analysis and close structural resemblance of DNA binding domains of AspBHI, PvuRts1I and AbaSI to the SRA domains of MspJI and eukaryotic proteins (Fig. 1C-D) suggest that these enzymes also flip-out the modified cytosine [18], [20]–[23].

However, for many eukaryotic 5(h)mC-binding proteins identified in the recent pull-down and mass-spectrometry studies [24], [25], the mechanism of modified cytosine recognition remains unknown. Mechanistic studies of these proteins would benefit from a fast solution-based method for the detection of extrahelical 5(h)mC. Here we used modification-dependent restriction endonucleases of MspJI and PvuRts1I families as a test system to assess the performance of several solution-based methods for extrahelical pyrimidine detection, including fluorescence measurements of the cytosine analog pyrrolocytosine and chemical display of extrahelical bases [26].

## Materials and Methods

### DNA oligonucleotides

Oligoduplex substrates used in this study are listed in Table 1. Oligonucleotides with 5-hydroxymethylcytosine modifications were purchased from IBA, all other oligonucleotides were from Metabion. Oligonucleotides were 5′-labeled with [γ-^33^P]ATP or [γ-^32^P]ATP (Hartmann Analytic) and T4 polynucleotide kinase (Thermo Fisher Scientific). Oligoduplexes were assembled by annealing the corresponding radiolabeled and unlabeled strands.

**Table 1:** Oligonucleotide substrates.

Duplex	Sequence a	Specification
16-M	5′-CGTAGC**M**TGGTCGATC-3′	
	3′-GCATCGGACCAGCTAG-5′	A short cognate LpnPI oligoduplex
16-C	5′-CGTAGCCTGGTCGATC-3′	
	3′-GCATCGGACCAGCTAG-5′	As 16-M but 5mC is replaced with an unmodified cytosine
16-P	5′-CGTAGC**P**TGGTCGATC-3′	
	3′-GCATCGGACCAGCTAG-5′	As 16-M but 5mC is replaced with a pyrrolocytosine
16-T	5′-CGTAGC**T**TGGTCGATC-3′	
	3′-GCATCG**G**ACCAGCTAG-5′	As 16-M but 5mC is replaced with a thymine
16-T-N	5′-CGTAGATCTTACGATC-3′	
	3′-GCATCTGGAATGCTAG-5′	Noncognate LpnPI oligoduplex with a T-G mismatch.
30-M	5′-CCGTAGC**M**TGGTCGATCCTAGCTGGTCGCC-3′	
	3′-GGCATCGGACCAGCTAGGATCGACCAGCGG-5′	An extended cognate LpnPI oligoduplex
30-C	5′-CCGTAGCCTGGTCGATCCTAGCTGGTCGCC-3′	
	3′-GGCATCGGACCAGCTAGGATCGACCAGCGG-5′	As 30-M but 5mC is replaced with an unmodified cytosine
30-P	5′-CCGTAGC**P**TGGTCGATCCTAGCTGGTCGCC-3′	
	3′-GGCATCGGACCAGCTAGGATCGACCAGCGG-5′	As 30-M but 5mC is replaced with a pyrrolocytosine
30-T	5′-CCGTAGC**T**TGGTCGATCCTAGCTGGTCGCC-3′	
	3′-GGCATCG**G**ACCAGCTAGGATCGACCAGCGG-5′	As 30-M but 5mC is replaced with a thymine
39-H/H	5′-CGACGATA**H**TTACCGGATAAGGCGCAATTAGATTACTTC-3′	
	3′-GCTGCTATGAATGGCCTATTCCGCGTTAAT**H**TAATGAAG-5′	Optimal YkrI and BmeDI substrate with two 5hmC-G base pairs
39-M/H	5′-CGACGATA**M**TTACCGGATAAGGCGCAATTAGATTACTTC-3′	
	3′-GCTGCTATGAATGGCCTATTCCGCGTTAAT**H**TAATGAAG-5′	As 39-H/H but one 5hmC is replaced with a 5-methylcytosine
39-C/H	5′-CGACGATA**C**TTACCGGATAAGGCGCAATTAGATTACTTC-3′	
	3′-GCTGCTATGAATGGCCTATTCCGCGTTAAT**H**TAATGAAG-5′	As 39-H/H but one 5hmC is replaced with an unmodified cytosine
39-P/H	5′-CGACGATA**P**TTACCGGATAAGGCGCAATTAGATTACTTC-3′	
	3′-GCTGCTATGAATGGCCTATTCCGCGTTAAT**H**TAATGAAG-5′	As 39-H/H but one 5hmC is replaced with a pyrrolocytosine
39-T/H	5′-CGACGATA**T**TTACCGGATAAGGCGCAATTAGATTACTTC-3′	
	3′-GCTGCTAT**G**AATGGCCTATTCCGCGTTAAT**H**TAATGAAG-5′	As 39-H/H but one 5hmC is replaced with a thymine
39-H	5′-CGACGATATTTACCGGATAAGGCGCAATTAGATTACTTC-3′	
	3′-GCTGCTATAAATGGCCTATTCCGCGTTAAT**H**TAATGAAG-5′	As 39-H/H but one 5hmC-G base pair is replaced with a T-A base pair
39-T	5′-CGACGATA**T**TTACCGGATAAGGCGCAATTAAATTACTTC-3′	
	3′-GCTGCTAT**G**AATGGCCTATTCCGCGTTAATTTAATGAAG-5′	As 39-T/H but the 5hmC-G base pair is replaced with a T-A base pair
31-C	5′-TGACCCACGCTCGCCCGGCGACACATTACGT-3′	
	3′-ACTGGGTGCGAGCGGGCCGCTGTGTAATGCA-5′	Cognate Ecl18kI oligoduplex with a C-G central base pair
31-M	5′-TGACCCACGCTCGCC**M**GGCGACACATTACGT-3′	
	3′-ACTGGGTGCGAGCGGGCCGCTGTGTAATGCA-5′	As 31-C but with a central 5mC-G base pair

a **M** designates 5-methylcytosine (5mC), **H** designates 5-hydroxymethylcytosine (5hmC), **P** designates pyrrolocytosine. Modified bases and mismatched base pairs are shown in boldface, recognition sequences are underlined.

### Proteins

The genes encoding LpnPI, YkrI, and BmeDI were amplified by PCR from the genomic DNA of *Legionella pneumophila* Philadelphia-1 (DSM No. 7513), *Yersinia kristensenii* (DSM No. 18543), and *Bacillus megaterium* (DSM No. 319), respectively. Genomic DNA was purchased from Leibnitz Institute DSMZ (Germany). Genes of LpnPI and its N-terminal DNA binding domain (LpnPI-N, corresponds to 1-224 residues of the full length protein) were cloned into the pLATE11 expression vector (Thermo Fisher Scientific). The first methionine in both proteins was replaced by a short hexahistidine tag (sequence MGHHHHHHG). Genes encoding YkrI and BmeDI were cloned into the pTYB2 expression vector as C-terminal fusions with the self-cleavable chitin binding domain. All proteins were expressed in the *E. coli* strain ER2566 (New England Biolabs). Cells were grown to OD_600_ 0.5–0.8 and induced with a final concentration of 0.2 mM IPTG at 16°C overnight, harvested by centrifugation and stored at −20°C.

The cells expressing LpnPI and LpnPI-N were sonicated in a buffer containing 20 mM Tris-HCl, pH 7.5, 450 mM NaCl, 10% v/v glycerol, and 7 mM 2-mercaptoethanol. Cleared lysates were collected after centrifugation at 40000 g for 1 h. Both proteins were purified by chromatography through HisTrap HP chelating and HiTrap Heparin HP columns and a gel-filtration column HiLoad 16/600 Superdex 200 pg (GE Healthcare).

YkrI and BmeDI were purified using a chitin column (New England Biolabs) as described by Wang et al. [15], and subsequently were loaded on a HiTrap Heparin HP column and eluted using a buffer containing Tris-acetate (pH 7.6) and 100–1000 mM potassium acetate.

Purified LpnPI and LpnPI-N were stored at −20°C in a buffer containing 20 mM Tris-HCl (pH 7.5 for LpnPI-N, pH 8.5 for LpnPI), 200 mM KCl, 1 mM DTT, and 50% v/v glycerol. The YkrI and BmeDI storage buffer contained 20 mM Tris-acetate (pH 7.5), 250 mM potassium-acetate, 1 mM DTT and 50% v/v glycerol.

Wt Ecl18kI was purified as described previously [27]. The purity of all proteins was higher than 95% as judged by SDS-PAGE. Protein concentrations were determined from *A_280_* measurements using the theoretical extinction coefficients calculated with the ProtParam tool available at http://web.expasy.org/protparam/. All protein concentrations are expressed in terms of monomer if not stated otherwise.

### Electrophoretic mobility shift assay

DNA binding was analysed by the electrophoretic mobility shift assay (EMSA) using ^33^P-labeled oligoduplexes. DNA (final concentration 1, 10 or 100 nM) was incubated with the protein (final concentrations varied from 5 to 1000 nM) for 15 min in 20 µl of the binding buffer containing either 40 mM Tris-acetate (pH 8.3 at 25°C) or 30 mM Mes-histidine (pH 6.3 at 25°C), 5 mM calcium-acetate, 0.1 mg/ml BSA and 10% v/v glycerol. Free DNA and protein–DNA complexes were separated by electrophoresis through 8% acrylamide gels (29∶1 acrylamide/bisacrylamide) in either 40 mM Tris–acetate, pH 8.3, or 30 mM Mes-histidine, pH 6.3, all with 5 mM calcium-acetate for 60-90 min at 5 V/cm. In some cases the binding and the electrophoresis buffers were devoid of calcium-acetate and instead were supplemented with 1 mM EDTA. Radiolabeled DNA and protein-DNA complexes were detected and quantified using the Cyclone phosphorimager and the OptiQuant software (Packard Instrument) [28].

### DNA cleavage experiments

DNA hydrolysis reactions were performed by manually mixing radiolabeled oligoduplexes (1 nM for YkrI and BmeDI, 400 nM for LpnPI) with the enzyme (100 nM YkrI and BmeDI, 500 nM LpnPI) in the Reaction Buffer (33 mM Tris-acetate, pH 8.0, 66 mM K-acetate, 10 mM Mg-acetate, 0.1 mg/ml BSA) at either 25°C (LpnPI) or 15°C (YkrI and BmeDI). Samples (8 µl) were collected at timed intervals and quenched by mixing with 12 µl of the loading dye solution (25 mM EDTA, pH 8.0, 95% v/v formamide, 0.01% bromphenol blue). Enzyme activity measurements at low pH were performed in a buffer containing 10 mM Mg-acetate, 50 mM Na-acetate, pH 4.3, and 0.1 mg/ml BSA. Reaction products were separated by denaturing polyacrylamide gel electrophoresis. The gels contained 20% 29∶1 acrylamide/bis-acrylamide with 8 M urea in standard tris-borate-edta (TBE) buffer, electrophoresis was performed for 1–2 h at 30 V/cm. Radiolabeled DNA was detected and quantified as described above. A single exponential was fitted to the substrate depletion data yielding the observed rate constant *k_obs_*. The *k_obs_* values were plotted as an average value from 2–4 experiments ±1 standard error.

### Pyrrolocytosine fluorescence measurements

Steady-state fluorescence measurements were acquired on a Fluoromax-3 (Jobin Yvon) spectrofluorimeter equipped with a Xe lamp. Sample temperatures were maintained at 25°C. Emission spectra (440–460 nm) were recorded at an excitation wavelength of 350 nm with excitation and emission bandwidths of 5 nm. The samples contained 1.0–2.0 µM of protein and 0.5 µM of pyrrolocytosine-labeled DNA oligoduplex (Table 1) in a buffer containing 30 mM Mes and 30 mM histidine (pH 6.3). Control spectra for the background correction were collected under identical conditions except that DNA lacking pyrrolocytosine modifications was used instead of the fluorescent DNA. The fluorescence emission value of the corrected spectrum was determined at 450 nm. The fluorescence intensity of the pyrrolocytosine-modified oligoduplex in the presence of the protein was divided by the fluorescence intensity in the absence of the protein. The resultant values (the fold change of pyrrolocytosine fluorescence upon addition of the protein) were plotted as an average value from several independent experiments ±1 standard error.

### Models of 5mC, 5hmC and pyC binding

The structures of the N-terminal domain of AspBHI (PDB ID 4oc8, chain A, residues 2-216), C-terminal domain of PvuRts1I (PDB ID 4oq2, chain A, residues 145–290) and the protein-DNA complex of UHRF1 SRA domain (PDB ID 3fde, chains ADE) where overlayed using Multiprot [29]. This procedure placed the 5mC base of the UHRF1-SRA DNA into the putative binding pockets of AspBHI and PvuRts1I (an overlay based on the MspJI-DNA structure, PDB ID 4r28, placed the 5mC base in a similar position). To remove minor steric clashes, the 5mC nucleotide in the AspBHI pocket was manually moved by 0.5 Å away from the R87 residue, by 1.0 Å from the Y83 residue and by 1.0 Å towards the D71 residue; in the PvuRts1I structure 5mC was moved by 1.1 Å away from the N217 residue and by 1.0 Å towards the W215 residue. In the resultant structures and in the structure of the McrBC DNA binding domain with methylated DNA (PDB ID 3ssc, chains ACD) the 5mC base was converted into a pyrrolocytosine residue using the ‘builder’ function of PyMOL (The PyMOL Molecular Graphics System, Version 1.4.1 Schrödinger, LLC), using 1.4 Å bond lengths for the C-C and C-N bonds in the 5-atom aromatic ring and a 1.5 Å C-C bond length for the extra-ring methyl group. A similar procedure yielded 5hmC base in the binding pocket of PvuRts1I.

### Reactions with CAA

DNA modification with chloroacetaldehyde (CAA) was performed as described by Daujotyte et al. [30]. Briefly, 100 nM radiolabeled DNA was mixed with 2 µM Ecl18kI, BmeDI or YkrI in 20 µl of the binding buffer (40 mM Tris-acetate, pH 8.3 at 25°C, 5% glycerol, 0.1 mg/ml BSA). Reactions were initiated by adding CAA to a final concentration of 0.5 M and were incubated for 1 h at 37°C. Modified strand cleavage was performed by adding 100 µl of freshly diluted 1 M piperidine and heating at 90°C for 30 min. DNA was precipitated with ethanol and resuspended in 8 µl of the loading dye solution (see above). DNA fragments were separated on high resolution denaturing polyacrylamide gels. The markers were generated using the standard A+G (formic acid) Maxam-Gilbert sequencing reactions.

### Chemical display of flipped out thymine and 5-methylcytosine

Experiments with thymine-substituted substrates were performed as described by Serva et al. [31]. Briefly, radiolabeled DNA (10 nM) and protein (100 nM) were mixed in the binding buffer (30 mM Mes-histidine, pH 6.3 at 25°C, 5% glycerol, 0.1 mg/ml BSA, total volume 20 µl). The reactions were initiated by adding KMnO_4_ to a final concentration of 2 mM, incubated for 5 min at 25°C and stopped by adding 20 µl of the solution containing 1.5 M Na-acetate (pH 7.0) and 1 M 2-mercaptoethanol. DNA was then precipitated with ethanol, redissolved in 1 M piperidine, heated at 90°C for 30 min, precipitated with ethanol, dissolved in 8 µl of the loading dye solution, and analyzed on a high-resolution denaturing polyacrylamide gel. 5-methylcytosine oxidation assay followed the same procedures, except that a 20 mM sodium-acetate reaction buffer (pH 4.3 at 25°C, ref. [32]) was used.

## Results

### Modification-dependent enzymes

**Figure 2:**
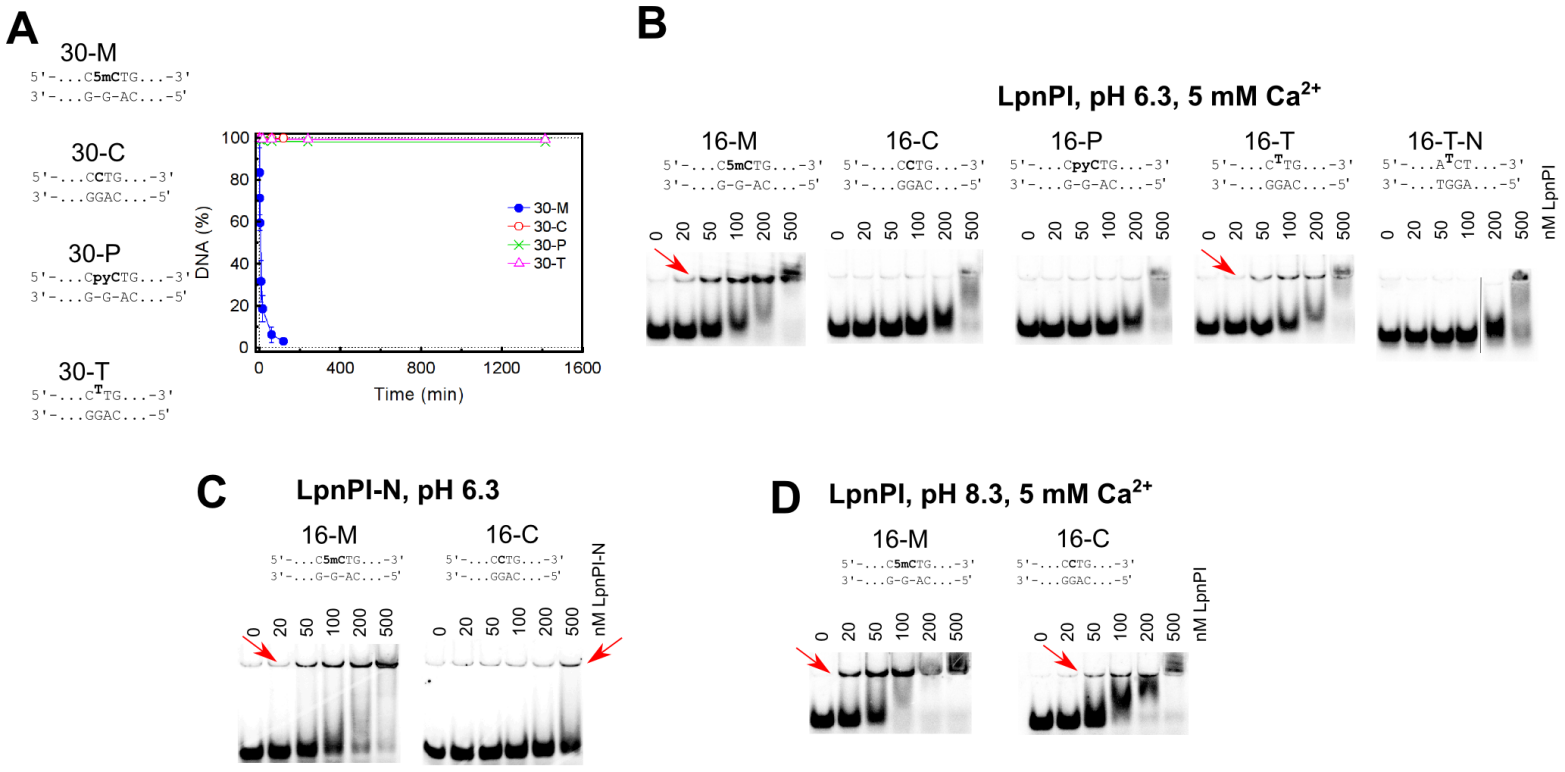
DNA cleavage and binding by LpnPI. The sequences in all panels depict recognition sites in the oligoduplex substrates. (A) DNA cleavage experiments. The reactions were performed with 500 nM enzyme (monomer) and 400 nM substrate at 25°C. Time courses of the reactions are shown. The reaction rate constant for the 30-M substrate equals 0.20±0.05 min^−1^. Reaction rate constants for other substrates were lower than 1×10^−5^ min^−1^. (B) Electrophoretic mobility shift assay with LpnPI. DNA binding experiments were performed in a pH 6.3 buffer in the presence of 5 mM Ca^2+^ ions. The final substrate concentration was 10 nM, LpnPI concentrations (in terms of monomer) are indicated above the gel lanes. Red arrows mark the location of the protein-DNA complexes. (C) Electrophoretic mobility shift assay with LpnPI DNA binding domain (LpnPI-N). Experiments with the cognate (16-M) and non-cognate (16-C) substrates were performed in a pH 6.3 buffer in the absence of Ca^2+^ ions. (D) Electrophoretic mobility shift assay with LpnPI in a pH 8.3 buffer in the presence of Ca^2+^ ions.

In this study we used three modification-dependent restriction endonucleases: LpnPI, YkrI and BmeDI. LpnPI belongs to the MspJI family and recognizes the DNA sequence 5′-CMDG-3′ (where M is 5mC or 5hmC, D – A, T or G) [33]. It is closely related to the structurally characterized enzyme AspBHI (>40% identical and ∼60% similar amino acid residues, S1A Figure), including nearly complete conservation of the presumed 5(h)mC binding pocket, (Fig. 1C). LpnPI cleaved the cognate oligoduplex 30-M with a rate constant of ∼0.2 min^−1^, but no cleavage was detected with an equivalent unmethylated oligoduplex 30-C (Fig. 2A). Discrimination between methylated and unmethylated DNA was also observed in electrophoretic mobility shift experiments (EMSA): both LpnPI and the N-terminal LpnPI DNA binding domain (LpnPI-N) formed protein-DNA complexes with methylated DNA at much lower protein concentrations than with unmethylated DNA (Fig. 2B,C). Noteworthy, the discrimination of specific (methylated) *vs* non-specific DNA by LpnPI was stronger at pH 6.3 and less pronounced at pH 8.3 (Fig. 2D). This indirectly supports the model of extrahelical 5mC recognition in the binding pockets of MspJI-like enzymes, which requires protonation of the conserved aspartate (D103 in MspJI, D71 in both AspBHI and LpnPI, Fig. 1B-C) [19], [22].

**Figure 3:**
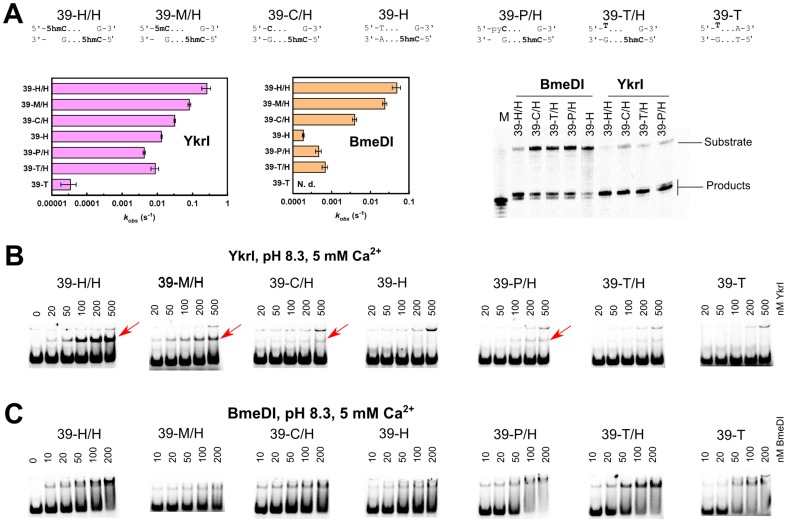
DNA cleavage and binding by YkrI and BmeDI. The sequences at the top of the figure schematically depict the 39-H/H (optimal substrate with two 5hmC bases), 39-M/H, 39-C/H, 39-H, 39-P/H, 39-T/H, and 39-T (one or both 5hmC-G base pairs replaced with a 5mC-G, C-G, T-A, pyrrolocytosine-G, and thymine-G base pairs, Table 1) oligoduplexes. (A) The observed first-order DNA cleavage rate constants. Cleavage reactions were performed with 1 nM substrate and 100 nM enzyme (monomer) at 15°C. In our experimental setup, BmeDI cleavage of the 39-T oligoduplex was not detectable. Denaturing PAGE analysis of cleavage products formed with various DNA substrates is shown on the right-hand side. Gel lane ‘M’ contained a synthetic single-stranded oligonucleotide that corresponds to cleavage of the bottom strand 11 nt downstream of the 5hmC nucleotide. (B) Electrophoretic mobility shift assay with YkrI. DNA binding experiments were performed in a pH 8.3 buffer in the presence of 5 mM Ca^2+^ ions. The final DNA concentration was 1 nM, and YkrI concentrations are indicated above the gel lanes. Red arrows mark the location of the specific YkrI-DNA complexes. The upper band corresponds to the low-mobility non-specific YkrI-DNA complex formed due to binding/aggregation of multiple protein molecules. (C) Electrophoretic mobility shift experiments with BmeDI. Reaction conditions were as in panel (B).

The YkrI and BmeDI display significant sequence similarities to the structurally characterized PvuRts1I-like family members PvuRts1I and AbaSI (S1B Figure). An optimal substrate for the PvuRts1I family enzymes consists of two 5hmC or 5ghmC nucleotides in the opposite DNA strands separated by a 20–22 bp DNA fragment [14], [15]. Current biochemical and structural data indicate that the 5(g)hmC sites are recognized by the DNA binding domains, while the two nuclease domains form a dimer and perform DNA cleavage at the center of the connecting DNA fragment, i. e. ∼11 nt from each modified base [21], [23]. Replacement of one 5hmC with a 5-methylcytosine, cytosine and a non-cytosine bases on a series of 39 bp substrates (oligoduplexes 39-H/H, 39-M/H, 39-C/H and 39-H respectively, Table 1) did not abolish their cleavage by YkrI and BmeDI, but decreased the reaction rate (Fig. 3A), suggesting that even a single DNA binding domain is enough to anchor the enzyme dimer to DNA via a 5hmC base. In this case the second YkrI/BmeDI DNA binding domain presumably makes contacts to the base located ∼20 bp downstream of the 5hmC nucleotide, and contributes to the enzyme-DNA complex stability depending on the structural similarity of the contacted base to 5(g)hmC. The cleavage data for YkrI is also complemented by EMSA experiments that show a gradual reduction in the amount of the specific enzyme-DNA complex as the second 5hmC in the optimal substrate is replaced with a 5-methylcytosine, cytosine and a non-cytosine base (Fig. 3B). However, we were unable to demonstrate such differences in binding for BmeDI (Fig. 3C).

### Pyrrolocytosine fluorescence measurements

Pyrrolocytosine (pyC) is a fluorescent cytosine analog that forms a stable base pair with guanine. The quantum yield of pyC fluorescence is sensitive to base unstacking [34], therefore pyC fluorescence measurements can be used to test the structural environment of a pyC base in nucleic acids and protein-DNA complexes [35], [36]. Notably, pyC fluorescence was used to confirm base flipping in solution by the DNA binding domain of the McrBC enzyme (McrB-N) [13]. However, similar experiments with PvuRts1I were inconclusive, as no increase in pyC fluorescence was observed, despite the fact that PvuRts1I cleaved the pyC-modified DNA [20].

An obvious prerequisite for binding and flipping of the pyC base is the ability of the enzyme to accommodate the flipped out base in the protein pocket. As this may be hindered due to the extra size of pyC in comparison to the unmodified cytosine or 5mC/5hmC bases, we first tested the ability of LpnPI, YkrI and BmeDI to bind and cleave pyrrolocytosine-modified DNA.

Upon replacement of 5mC with a pyC (substrate 16-P), the LpnPI and LpnPI-N binding to DNA became indistinguishable from the unmethylated DNA (Fig. 2B). The same was true for the pyC DNA cleavage (Fig. 2A). For YkrI and BmeDI we used a derivative of the optimal substrate with one 5hmC replaced with a pyC (substrate 39-P/H, Table 1). DNA binding and cleavage experiments performed with both enzymes indicated that the replacement of one 5hmC with a pyC compromises enzyme binding and activity to a similar extent as the replacement with an unmodified cytosine or a non-cytosine base (Fig. 3). We therefore conclude that pyC is a poor 5mC/5hmC substitute for LpnPI, YkrI and BmeDI. Indeed, modeling of the pyC base into the presumed binding pocket of AspBHI and PvuRts1I, preserving the H-bonding interactions with the conserved polar pocket residues, results in steric clashes (S2A Figure). Not surprisingly, none of the proteins used in our study (LpnPI, YkrI and BmeDI) triggered fluorescence change of the pyC-containing DNAs (S2B Figure). In contrast, almost no clashes are observed when pyC is modeled into the pocket of the DNA binding domain of McrBC (S2A Figure), which readily binds pyC DNA and extrudes the modified base from the double helix [13].

### Reactions with chloroacetaldehyde

Chloroacetaldehyde (CAA) is known to react with unpaired cytosine and adenine bases in DNA yielding 3, N4-ethenocytosine and 1, N6-ethenoadenine [37]. Such modified residues can be detected by piperidine-induced strand cleavage. So far, the suitability of the CAA reaction was demonstrated for mapping of unmodified cytosine flipped out by several DNA cytosine-5 methyltransferases and restriction enzymes [30]. Since CAA also reacts with 5-methylcytosine [38], we asked if the same experimental setup could be used to detect extrahelical 5mC. As a control we used endonuclease Ecl18kI. This base-flipping restriction enzyme recognizes the pseudosymmetric DNA site 5′-CCNGG-3′ and flips out the nucleotides of the central base pair [39] that become sensitive to CAA modification [30]. Ecl18kI binds DNA oligoduplexes with the central C-G and 5mC-G base pairs with comparable affinity both in the absence and in the presence of CAA (S3A Figure). Nevertheless, enhanced DNA cleavage in the Ecl18kI complex after CAA/piperidine treatment was observed only for the unmodified central cytosine, but not for 5-methylcytosine (S3B Figure). Thus, at least under standard reaction conditions used in our study, CAA can not be used to detect extrahelical 5mC. Therefore, we could only test if YkrI and BmeDI flip out the unmodified cytosine from the suboptimal substrate 39-C/H, which contains an unmodified cytosine base located ∼20 bp away from the 5hmC base (Table 1). We rationalized that while one DNA binding domain of the dimeric enzyme is engaged in a high affinity interaction with the 5hmC site, the second DNA binding domain may interrogate the base ∼20 bp downstream, in this case a cytosine, and this process may involve base flipping. This is supported by the observation that both YkrI and BmeDI cleave the 39-C/H substrate faster than the 39-H substrate, which lacks a cytosine base ∼20 nt downstream of the 5hmC (Fig. 3). However, neither YkrI nor BmeDI increased the reactivity of the target cytosine in the 39-C/H duplex with CAA (data not shown). Among other reasons for the lack of cytosine reactivity (no flipping, insufficient life-time of the flipped out base, inactivation of the enzyme due to CAA treatment) is the mechanism for the extrahelical 5mC/5hmC recognition by the SRA domains. In the solved co-crystal structures of the UHRF1 SRA domain and the MspJI REase, the Watson-Crick edge of the flipped out 5mC makes hydrogen bonds to the pocket residues (Fig. 1A-B) [8]–[12], [19]. Conserved residues capable of hydrogen-bonding interactions with the Watson-Crick edge of cytosine derivatives are also present in both PvuRts1I (N217, E228, Fig. 1C) and AbaSI (N236, E247), and are conserved in YkrI/BmeDI (S1B Figure), suggesting that a cytosine base, had it been extruded from the DNA double helix, would be shielded from CAA due to the hydrogen-bonding interactions with the protein. In sharp contrast, Ecl18kI flips out both purine and pyrimidine bases, and binds them in a cavernous protein pocket without forming any base-specific contacts [39]; this may explain the efficiency of CAA modification of the extrahelical cytosine in the Ecl18kI-DNA complex [30].

### Permanganate oxidation of extrahelical pyrimidines

Under acidic conditions potassium permanganate oxidizes both thymine and 5-methylcytosine [32]. However, at physiologic pH this reaction is limited primarily to thymine. KMnO_4_ treatment leads to conversion of pyrimidine bases to 5,6-dihydroxy-5,6-dihydropyrimidines [40]; the oxidized bases undergo further degradation leading to cleavage of the phosphodiester backbone upon piperidine treatment. Since the oxidation reaction of the C5 = C6 bond requires an access to the side of pyrimidine ring that is hidden in the double-stranded DNA, thymines and 5-methylcytosine in DNA helix are relatively resistant to permanganate oxidation compared to extrahelical pyrimidines. KMnO_4_ was used to detect flipped-out thymines for cytosine and adenine DNA methyltransferases and a sequence-specific transposase [31], [41], [42]. We asked if the permanganate oxidation assay could help detect base-flipping by the modification-dependent restriction enzymes.

Since the KMnO_4_ assay at near-neutral pH works only with the thymine bases, we made 5mC/5hmC to thymine replacements in the standard LpnPI and PvuRts1I family substrates, thereby creating oligoduplexes with T-G mispairs (Table 1). EMSA experiments confirmed that LpnPI specifically binds the T-G mismatch substrate 16-T, albeit less tightly than the standard methylated duplex 16-M (Fig. 2B). However, we were unable to detect any T-substituted substrate cleavage by LpnPI, both in the standard reaction buffer (Fig. 2A) and under conditions mimicking the EMSA experiment (data not shown). On a control oligoduplex containing the T-G mismatch in a different sequence context (oligoduplex 16-T-N), we observed neither specific binding nor cleavage (Fig. 2B). Replacement of a single 5hmC base in the optimal YkrI/BmeDI oligoduplex 39-H/H with a thymine (substrate 39-T/H, Table 1) decreased the binding and cleavage of the substrate to a similar extent as the 5hmC-to-cytosine or the 5hmC-to-non-cytosine replacements (substrates 39-C/H and 39-H, Fig. 3A-B), but did not change the cleavage position (BmeDI cleaves all substrates 11-12 nt, YkrI – 12 nt downstream from 5hmC, Fig. 3A); no YkrI binding was observed with the ‘non-cognate’ thymine-substituted oligoduplex 39-T (Fig. 3B). Taken together, the T-G mismatch is a poor substitute for a normal 5(h)mC-G base pair for all enzymes used in our study. A primary reason for this presumably is the direct read-out of the target base: the pockets for extrahelical base binding in the SRA domains, and their homologs in the MspJI/PvuRts1I-like enzymes, are optimized for the specific hydrogen-bonding interactions with cytosine derivatives, but not thymine (Fig. 1).

**Figure 4:**
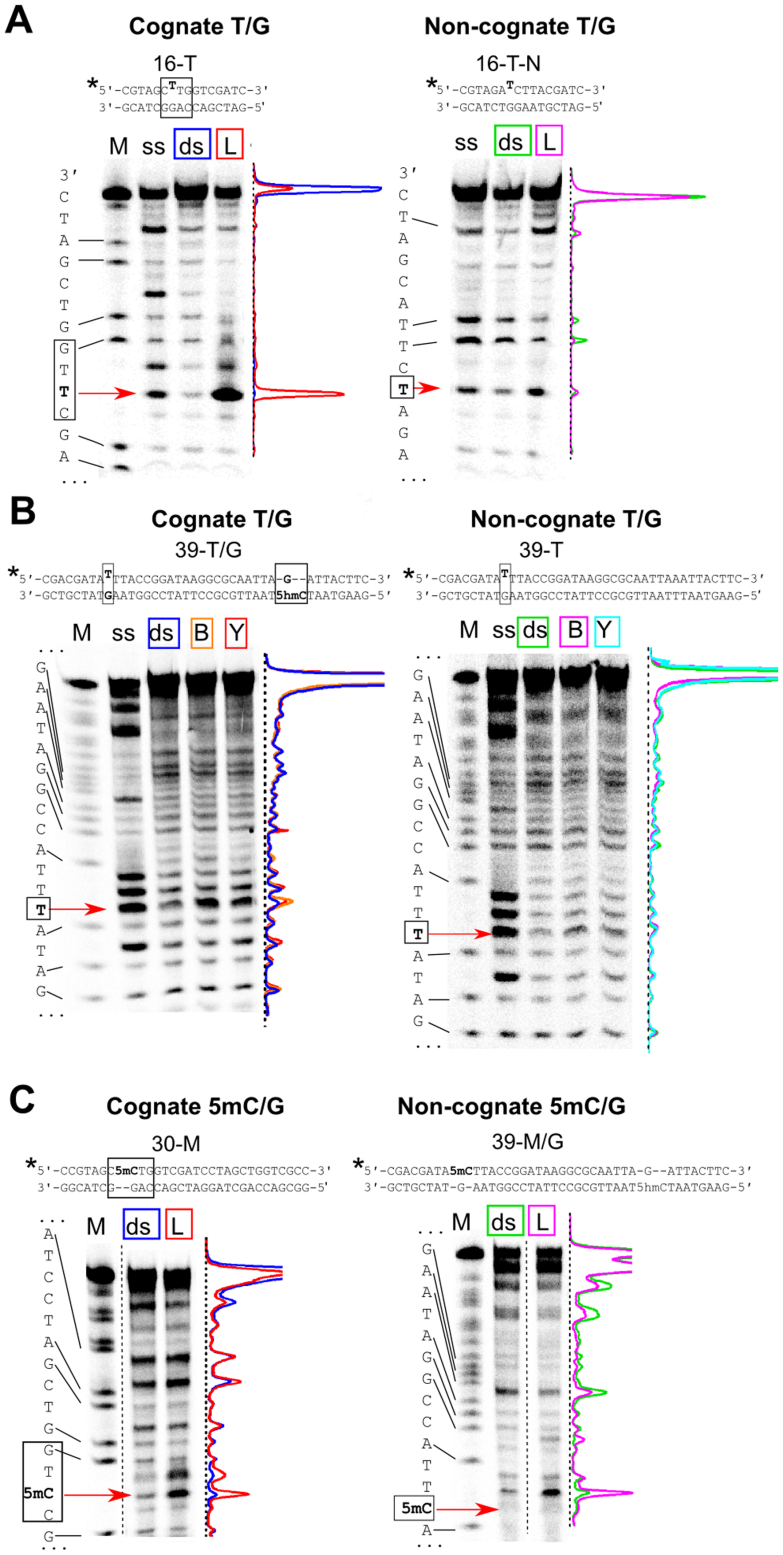
Permanganate reactivity of pyrimidine bases in the protein-DNA complexes. Sequences at the top of the panels schematically depict the ‘cognate’ and the ‘non-cognate’ thymine-substituted substrates, the asterisk marks the ^33^P radiolabel. Base pairs important for specific binding are in black boxes. Positions of the mispaired thymine and the 5-methylcytosine are marked with red arrows. (A) Thymine oxidation by KMnO_4_ with or without LpnPI-N. ‘M’, the A+G markers of the radiolabeled ‘cognate’ and ‘non-cognate’ strands; ‘ss’, oxidation of the single-strand oligonucleotides; ‘ds’, double-stranded 16-T (cognate) and 16-T-N (noncognate) oligoduplexes without the protein; ‘L’, 16-T and 16-T-N oligoduplexes + LpnPI-N. Density profiles of individual lanes are shown: cognate DNA (blue), cognate DNA + LpnPI-N (red), non-cognate DNA (green), and non-cognate DNA + LpnPI-N (magenta). (B) Thymine oxidation by KMnO_4_ in the presence of YkrI and BmeDI. ‘M’, the A+G markers of the radiolabeled ‘cognate’ and ‘non-cognate’ strands; ‘ss’, oxidation of the single-strand oligonucleotides; ‘ds’, 39-T/H (cognate) and 39-T (noncognate) oligoduplexes without the protein; ‘Y’, 39-T/H and 39-T oligoduplexes + YkrI; ‘B’, 39-T/H and 39-T oligoduplexes + BmeDI. Density profiles: cognate DNA (blue), cognate DNA + BmeDI and YkrI (orange and red, respectively), non-cognate DNA (green), non-cognate DNA + BmeDI and YkrI (magenta and cyan, respectively). (C) 5-methylcytosine oxidation by KMnO_4_ at pH 4.3 with or without LpnPI-N. ‘M’, the A+G markers of the radiolabeled ‘cognate’ and ‘non-cognate’ strands; ‘ds’, double-stranded 30-M (cognate) and 39-M/H (noncognate) oligoduplexes without the protein; ‘L’, 30-M and 39-M/H oligoduplexes + LpnPI-N. Density profiles of individual lanes are colored as in panel (A).

Surprisingly, incubation of the T-substituted substrates with LpnPI-N, LpnPI, YkrI, and BmeDI significantly increased the susceptibility of the mispaired thymine to KMnO_4_ oxidation. A particularly strong enhancement in reactivity was observed with the ‘cognate’ mismatch substrate 16-T and LpnPI-N (Fig. 4A). Since the increase was not detectable with the ‘non-cognate’ T-G oligoduplex 16-T-N, we attribute the hyper-reactivity of the target thymine to the change in its environment induced by the specific binding of the modification-dependent enzyme. The increase in thymine reactivity of the ‘cognate’ T-G substrate 39-T/H upon its incubation with YkrI and BmeDI was less pronounced, but still clearly detectable (Fig. 4B). Almost no hyper-reactivity of the mispaired thymine was observed with the ‘non-cognate’ T-G oligoduplex 39-T (Fig. 4B), again implying that the change in the mispaired thymine environment observed with the ‘cognate’ thymine-substituted DNA was due to the specific enzyme interaction with the DNA.

The major drawback of the above assay is that it makes use of a mutated substrate. To strengthen the evidence for native modified cytosine flipping, we also performed KMnO_4_ reactions on native 5mC-containing substrates. However, the KMnO_4_ reacts with 5mC only at non-physiological pH (4.3), where the protonation state of both protein and DNA bases may interfere with protein function and the stability of the protein-DNA complex (nevertheless, examples of DNA-protein interaction studies performed at a very wide range of pH values are present in literature, e. g., ref. [43]). Though none of the enzymes used in our study showed any catalytic activity at pH 4.3, incubation of LpnPI-N with the cognate substrate 30-M, containing 5mC in the context of the LpnPI recognition site (Table 1), resulted in a significant enhancement of 5mC sensitivity to permanganate (Fig. 4C). This signal seems to be both LpnPI- and 5mC-specific, as control experiments performed on the 39-M/H oligoduplex containing 5mC in a different sequence context showed no enhancement in 5mC reactivity (though increase in reactivity was observed for some thymine residues, Fig. 4C). When the 39-M/H oligoduplex (contains one 5mC and one 5hmC separated by 21 bp, Table 1), was incubated with YkrI and BmeDI, no changes in 5mC reactivity were observed (S4 Figure). Taken together, permanganate reactions are consistent with thymine (both LpnPI and YkrI/BmeDI) and 5-methylcytosine (LpnPI) flipping by the cytosine modification-dependent restriction enzymes, but the results are obtained either on a mutated substrate or at non-physiological pH.

## Discussion

In the present study we employed three established fluorescence and chemical-display based methods to test base flipping by the modification-dependent restriction endonucleases. Our results indicate that each assay has its strengths and limitations, and neither of them suits all 5mC/5hmC-binding proteins. For example, the replacement of the target base with the fluorescent base pyrrolocytosine is a convenient method to detect base flipping by measuring the changes in pyrrolocytosine fluorescence intensity upon protein binding. In particular, it was successfully applied for the study of the modification-dependent enzyme McrBC [13]. However, for the method to work, the protein in question must have an adequately sized protein pocket to accommodate the bulky pyrrolocytosine base. While this seems to be the case with the DNA binding domain of McrBC, modeling, DNA binding and cleavage studies indicate that the SRA-like domains of MspJI- and PvuRts1I-like enzymes used in our study do not tolerate such a replacement (Figs. 2, 3 and S2 Figure). The second assay tested in our study, chloroacetaldehyde modification [30], does not require non-natural base substitutions, but under conditions used in our study it worked only with unmodified cytosine (S3 Figure).

The third base-flipping assay tested in our study makes use of the hyper-sensitivity of extrahelical pyrimidines to KMnO_4_ oxidation. At near-neutral pH this method required replacement of the 5mC/5hmC bases with thymines, thereby forming T-G mismatches. Due to the perturbed geometry of the mismatched base pairs, the unpaired thymines can themselves become hyper-reactive [44]. Fortunately, relatively low background signal observed in our experiments indicates that the accessibility of the mismatched thymines to the bulk solvent is limited, even though they are flanked from both sides with pyrimidine bases (oligoduplexes 16-T and 39-T/H, Table 1). Though similar in size to both 5mC and 5hmC, thymine was poorly tolerated by the modification-dependent enzymes used in our study, resulting in impaired binding and cleavage of the thymine-substituted substrates (Figs. 2 and 3). Surprisingly, incubation of the thymine-substituted DNA with these proteins resulted in hyper-sensitivity of the mismatched thymine to KMnO_4_ oxidation. A potential risk of using a mismatched oligoduplex is that the mismatch may induce additional conformational flexibility at or in the vicinity of the mispair that upon binding of a protein may result in stronger conformational changes as compared with those in a standard double-stranded DNA. However, the increase in reactivity in our experiments was localized to the target thymine, and was observed only with the ‘cognate’ thymine-substituted substrates (Fig. 4), implying formation of native-like protein-DNA complexes with a flipped-out thymine.

Structural studies and modeling suggest that the flipped-out base in the protein pocket of the SRA-like domains is sandwiched between conserved polar, aromatic and hydrophobic residues (Fig. 1). If the mispaired thymine occupies the same position as the 5mC/5hmC residues, it should be shielded from KMnO_4_ oxidation. Instead, the hyper-reactivity of the thymines suggests that the thymine base may be in a dynamic equilibrium between the intra-helical state and an ensemble of flipped-out states. Complete flipping and trapping of the thymine in the protein pocket, most likely, is hindered by the failure of thymine to form hydrogen bonds with the pocket residues that are tailored for the direct read-out of the cytosine derivatives (Fig. 1); on the other hand, opening of the T-G mismatch is much more easily achieved than the disruption of the native C-G base pair, thereby shifting the equilibrium towards the extrahelical states. Interestingly, the recently solved co-crystal structure of the MspJI-DNA complex [19] revealed both specific base-flipping of a 5mC base by one DNA recognition domain, and non-specific flipping of a guanine residue by another DNA binding domain. In the latter case the extruded base occupies a slightly different position than 5mC, and the binding pocket remains in the more open conformation. Formation of such complex was interpreted as a possible target site search intermediate [19]. The extruded thymine detected in our study with LpnPI/YkrI/BmeDI could also occupy a similar non-native position that would permit the reaction with KMnO_4_.

To strengthen the evidence for native modified cytosine flipping by the modification-dependent enzymes, we also probed the permanganate oxidation of 5mC bases at pH 4.3. Due to non-native conditions, this method was previously applied only for detection of 5mC in DNA [32]. Surprisingly, this assay revealed a significant sequence- and 5mC-specific ‘positive’ signal for LpnPI, which is consistent with 5mC flipping by this enzyme (whether the flipped-out base at such pH occupies the same position as in the native complex remains unknown). No signal was observed with YkrI and BmeDI, the most likely reason for the lack of the signal being the non-physiological pH, which may interfere with DNA binding by most proteins. Nevertheless, the KMnO_4_ assay at low pH may work with some proteins, and therefore can prove useful in the studies of the 5mC recognition mechanism of other modified cytosine ‘readers’.

Taken together, our study demonstrates the usefulness and limitations of several solution-based methods for the detection of flipped-out cytosine and its derivatives. Only one of the tested methods – permanganate oxidation of the extrahelical pyrimidines – provided evidence for base flipping by the modification-dependent restriction enzymes, implying that the lack of the ‘positive signal’ in one or even several assays does not exclude base flipping. Therefore, the DNA recognition mechanism of potential 5mC/5hmC-binding proteins should be tested using a combination of all available methods. Nevertheless, the final proof or disproof for the base flipping mechanism would still require high resolution structures of protein-DNA complexes.

## Supporting Information

S1 Figure **Modification-dependent endonucleases used in the study.** (A) Alignment of the MspJI family member LpnPI with the structurally characterized enzyme AspBHI. Numbering of AspBHI secondary structure elements is taken from [22]. (B) Alignment of the PvuRts1I family members YkrI and BmeDI with the structurally characterized enzymes AbaSI and PvuRts1I. Numbering of AbaSI secondary structure elements is taken from [21]. In both panels green squares mark residues forming the walls of the putative flipped-out base binding pocket; black triangles mark pocket residues that are predicted to contact the Watson-Crick edge of the flipped-out base; stars mark the catalytic centers. The figure was generated with ESPript [46].
(TIF)

S2 Figure **Experiments with pyrrolocytosine-substituted DNA.** (A) The models of pyrrolocytosine base in the protein binding pockets of AspBHI, PvuRts1I, and the DNA binding domain of McrBC (see Materials and Methods for details). The black and green lines mark the boundaries of the protein pockets (cut at the plane of the cytosine ring) and the pyrrolocytosine base, respectively. Only the McrBC domain accommodates the pyrrolocytosine base without steric clashes. (B) Pyrrolocytosine fluorescence measurements performed with LpnPI, YkrI and BmeDI. The graphs show the ratio of the cognate pyrrolocytosine-substituted oligoduplex (16-P for LpnPI and LpnPI-N, 39-P/H for YkrI and BmeDI) fluorescence intensity in the presence of the protein to the fluorescence intensity of the same oligoduplex in the absence of the protein.
(TIF)

S3 Figure **The chloroacetaldehyde modification assay with Ecl18kI restriction enzyme.** Sequences at the top of the panel A schematically depict the 31-C (central base pair C-G) and 31-M (central base pair 5mC-G) oligoduplexes; the asterisk marks the radiolabel. (A) Ecl18kI binding to DNA oligoduplexes 31-C and 31-M in the pH 8.3 binding buffer in the presence of 5 mM Ca^2+^. Final DNA concentration was 100 nM. Samples in gel lanes ‘C’ contained 1000 nM enzyme (dimer) and 500 mM CAA. Red arrows mark the position of the specific protein-DNA complexes. (B) DNA modification with CAA in the presence and in the absence of Ecl18kI. Red arrows mark the position of the central cytosine or 5-methylcytosine. Lanes ‘M’, the A+G markers of the radiolabeled strands; ‘ds’, 31-C (unmodified cytosine) and 31-M (5mC) oligoduplexes without the protein; ‘E’, 31-C and 31-M oligoduplexes + Ecl18kI. The normalized density profiles of individual lanes are shown at the bottom of the panel: ‘ds’ (blue), ‘E’ (red).
(TIF)

S4 Figure **5-methylcytosine oxidation by KMnO_4_ at pH 4.3 with or without YkrI and BmeDI.** Sequence at the top of the image schematically depict the substrate, the asterisk marks the ^33^P radiolabel. Base pairs 5mC-G and 5hmC-G important for specific binding are in black boxes. Position of the 5-methylcytosine in the autoradiograph is marked with a red arrow. ‘M’, the A+G marker of the radiolabeled substrate strand; ‘ds’, double-stranded 39-M/H oligoduplex without the protein; ‘Y’, 39-M/H oligoduplex + YkrI; ‘B’, 39-M/H oligoduplex + BmeDI. The normalized density profiles of individual lanes are shown: 39-M/H DNA (blue), 39-M/H DNA + BmeDI and YkrI (orange and red, respectively).
(TIF)
