# Supplementary figures and images for: Chemical Display of Pyrimidine Bases Flipped Out by Modification-Dependent Restriction Endonucleases of MspJI and PvuRts1I Families
Source: PLoS One. 2014 Dec 8;9(12):e114580. doi: 10.1371/journal.pone.0114580 (PMC4259335; doi:10.1371/journal.pone.0114580)

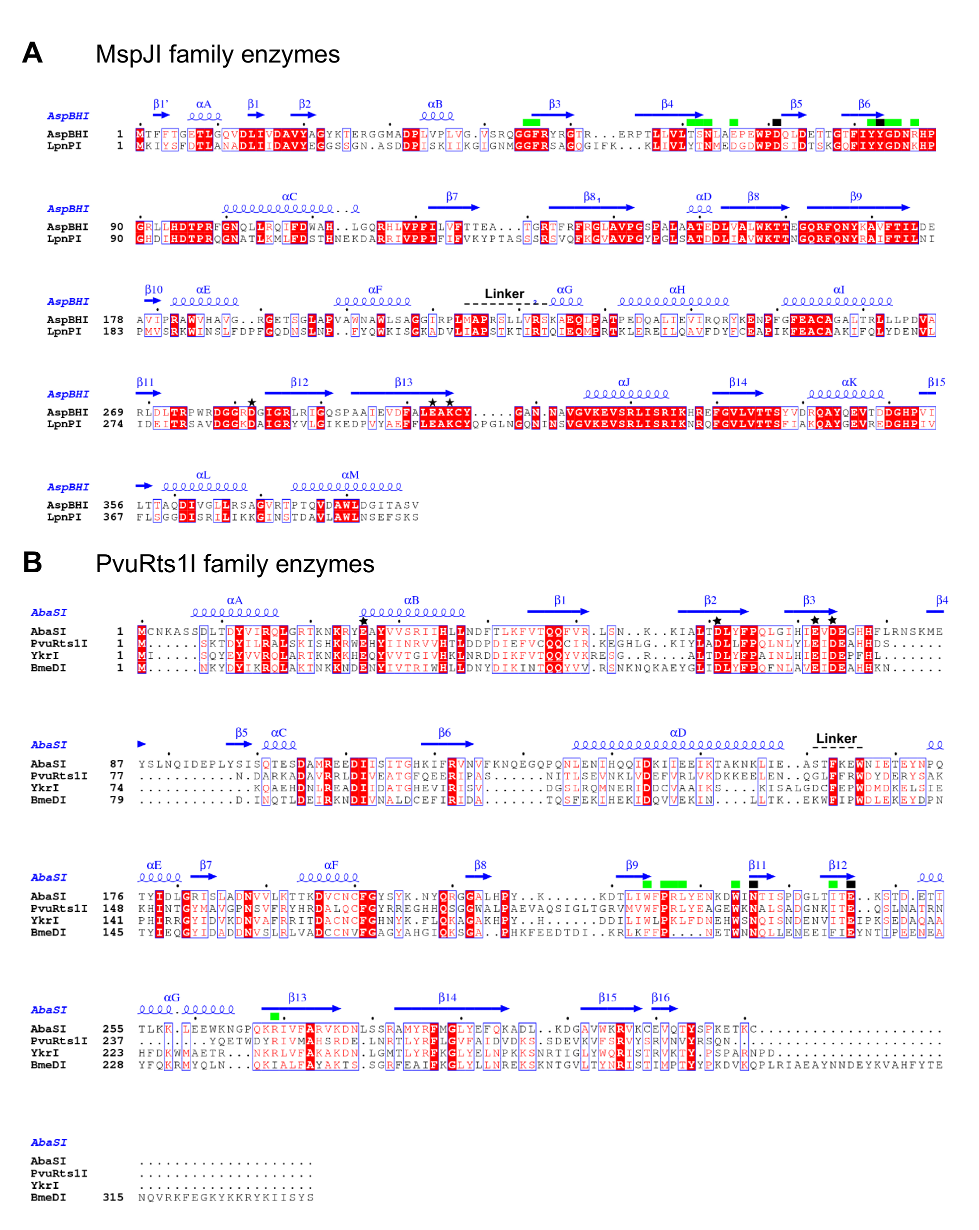

Supplement: S1 Figure — Modification-dependent endonucleases used in the study. (A) Alignment of the MspJI family member LpnPI with the structurally characterized enzyme AspBHI. Numbering of AspBHI secondary structure elements is taken from [22]. (B) Alignment of the PvuRts1I family members YkrI and BmeDI with the structurally characterized enzymes AbaSI and PvuRts1I. Numbering of AbaSI secondary structure elements is taken from [21]. In both panels green squares mark residues forming the walls of the putative flipped-out base binding pocket; black triangles mark pocket residues that are predicted to contact the Watson-Crick edge of the flipped-out base; stars mark the catalytic centers. The figure was generated with ESPript [46]. (TIF) [file pone.0114580.s001.tif]

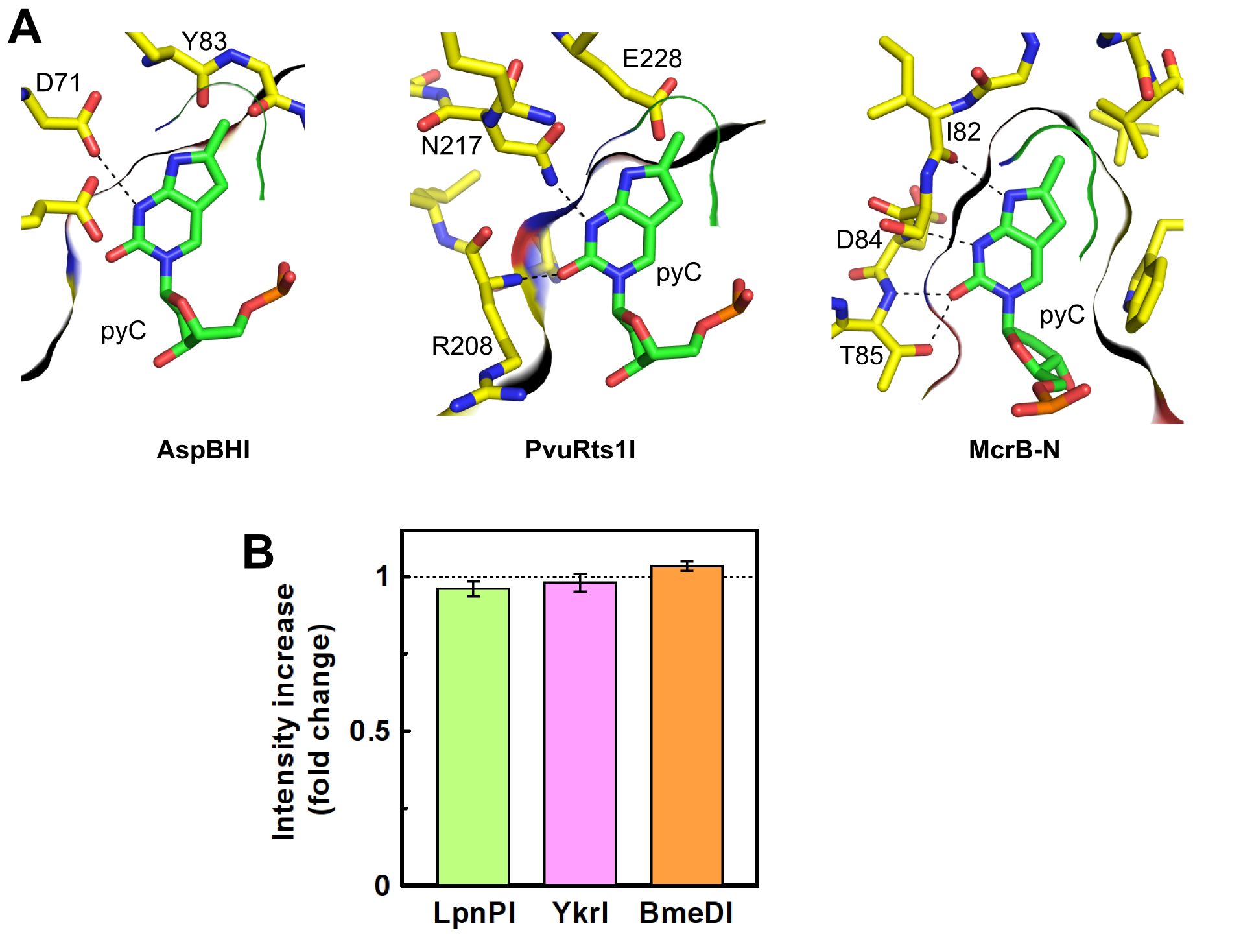

Supplement: S2 Figure — Experiments with pyrrolocytosine-substituted DNA. (A) The models of pyrrolocytosine base in the protein binding pockets of AspBHI, PvuRts1I, and the DNA binding domain of McrBC (see Materials and Methods for details). The black and green lines mark the boundaries of the protein pockets (cut at the plane of the cytosine ring) and the pyrrolocytosine base, respectively. Only the McrBC domain accommodates the pyrrolocytosine base without steric clashes. (B) Pyrrolocytosine fluorescence measurements performed with LpnPI, YkrI and BmeDI. The graphs show the ratio of the cognate pyrrolocytosine-substituted oligoduplex (16-P for LpnPI and LpnPI-N, 39-P/H for YkrI and BmeDI) fluorescence intensity in the presence of the protein to the fluorescence intensity of the same oligoduplex in the absence of the protein. (TIF) [file pone.0114580.s002.tif]

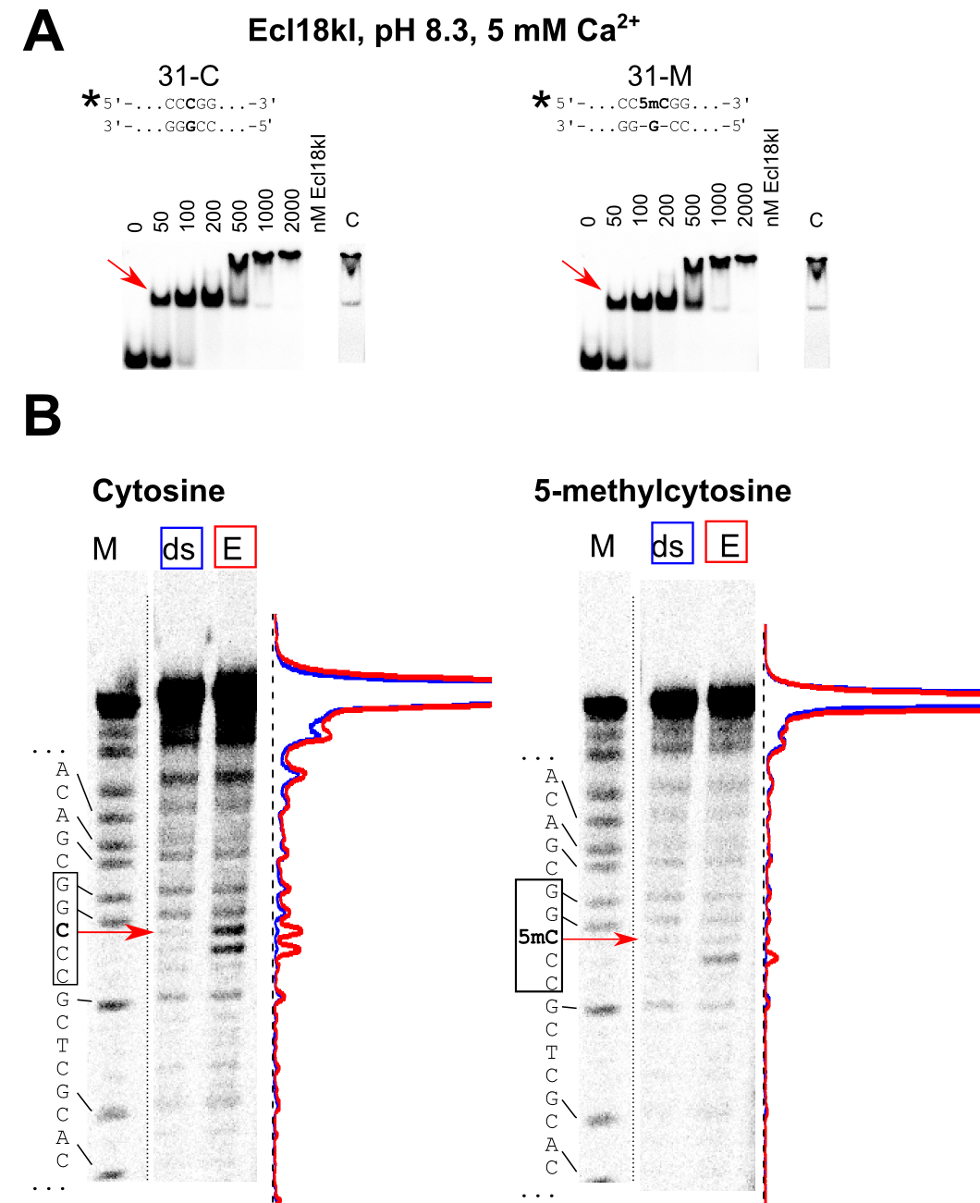

Supplement: S3 Figure — The chloroacetaldehyde modification assay with Ecl18kI restriction enzyme. Sequences at the top of the panel A schematically depict the 31-C (central base pair C-G) and 31-M (central base pair 5mC-G) oligoduplexes; the asterisk marks the radiolabel. (A) Ecl18kI binding to DNA oligoduplexes 31-C and 31-M in the pH 8.3 binding buffer in the presence of 5 mM Ca2+. Final DNA concentration was 100 nM. Samples in gel lanes ‘C’ contained 1000 nM enzyme (dimer) and 500 mM CAA. Red arrows mark the position of the specific protein-DNA complexes. (B) DNA modification with CAA in the presence and in the absence of Ecl18kI. Red arrows mark the position of the central cytosine or 5-methylcytosine. Lanes ‘M’, the A+G markers of the radiolabeled strands; ‘ds’, 31-C (unmodified cytosine) and 31-M (5mC) oligoduplexes without the protein; ‘E’, 31-C and 31-M oligoduplexes + Ecl18kI. The normalized density profiles of individual lanes are shown at the bottom of the panel: ‘ds’ (blue), ‘E’ (red). (TIF) [file pone.0114580.s003.tif]

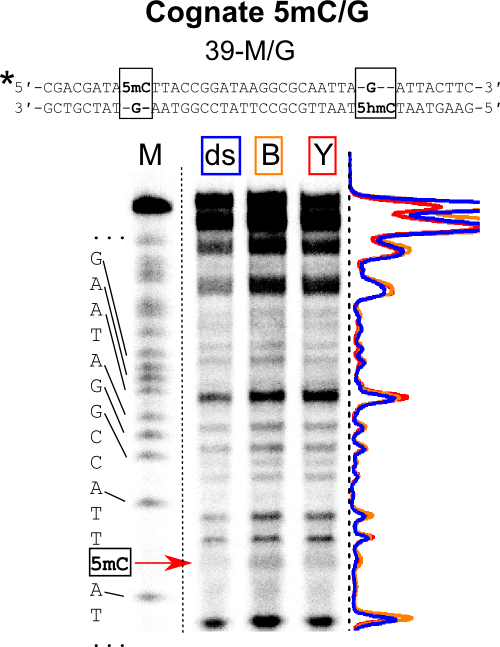

Supplement: S4 Figure — 5-methylcytosine oxidation by KMnO4 at pH 4.3 with or without YkrI and BmeDI. Sequence at the top of the image schematically depict the substrate, the asterisk marks the 33P radiolabel. Base pairs 5mC-G and 5hmC-G important for specific binding are in black boxes. Position of the 5-methylcytosine in the autoradiograph is marked with a red arrow. ‘M’, the A+G marker of the radiolabeled substrate strand; ‘ds’, double-stranded 39-M/H oligoduplex without the protein; ‘Y’, 39-M/H oligoduplex + YkrI; ‘B’, 39-M/H oligoduplex + BmeDI. The normalized density profiles of individual lanes are shown: 39-M/H DNA (blue), 39-M/H DNA + BmeDI and YkrI (orange and red, respectively). (TIF) [file pone.0114580.s004.tif]
